# Long-Term Clinical Performance of Aesthetic Restorations in Primary Molars: A Case Report

**DOI:** 10.1155/2011/515713

**Published:** 2011-09-07

**Authors:** Luciana Pomarico, Beatriz Gonçalves Neves, Lucianne Cople Maia, Laura Guimarães Primo

**Affiliations:** ^1^Faculty of Dentistry, Fluminense Federal University, Nova Friburgo, RJ 28652-650, Brazil; ^2^Department of Pediatric Dentistry and Orthodontics, Faculty of Dentistry, Federal University of Rio de Janeiro, Rio de Janeiro, RJ 21941-913, Brazil

## Abstract

There is a great diversity of restorative materials and techniques for deciduous molars with significant coronal destruction, including resin composite restorations and biologic restorations (portions of natural teeth). By using 4 evaluation methods, this study aimed at longitudinally evaluating the effectiveness of restorations in the deciduous molars of a patient having high caries activity, using adhesive techniques. The evaluation methods consisted of the fibre-optic transillumination method, clinical evaluation based on the United States Public Health Service criteria, radiographs, and an indirect method, scanning electron microscopy. Despite the patient's poor biofilm control, the restorative techniques were shown to be efficacious, particularly the biologic restorative technique.

## 1. Introduction

Restorative techniques for deciduous teeth are still an important aspect of pediatric dentistry since caries remains a significant oral health problem.In the face of imminent dental restoration, some factors should be taken into account before choosing the best restorative material. First, clinical conditions should be evaluated to determine whether the treatment will be emergency (temporary) or definitive. Tooth longevity, aesthetic need, family's financial resources [1], the patient's behaviour—an important aspect of children's dentistry [2]—and the patient's caries risk should also be considered.

For many years, decayed deciduous molars were restored with amalgam [3] or, in cases involving large cavities, stainless steel crowns [4]. More recently, adhesive composite materials have emerged as a way to reinforce the remaining dental structure, promote better marginal adaptation, and improve aesthetics [1, 5–7]. Another option is biologic restoration, which uses natural dental crowns obtained through a tooth bank for restoring decayed teeth. This technique not only achieves high anatomic and aesthetic quality but also ensures that natural color and surface smoothness are preserved [8], once it uses natural teeth. In “tooth banks” temporary teeth are organized by their group (incisors, canines, and molars), and they are achieved in accordance with professional's need. In such cases, the professional chooses the preferred tooth considering also its mesiodistal distance that must be as similar as possible to the reminiscent space to be restored.

Regardless of the restorative material or technique used, the main problem faced by practitioners using adhesive restorations is the risk of microleakage [9], especially in patients presenting high caries activity, poor oral hygiene, and irregular dietary habits.

Amongst the methods of evaluating the efficiency of such restorative treatments are fibre-optic transillumination (FOTI) method, clinical criteria, radiographs, and indirect methods such as scanning electron microscopy (SEM). The first method consists of using a white-light source for detecting the presence of microleakage and secondary caries and for examining the tooth-restoration interface, since sound dental structures have a light-emitting index different from that of restorative material [10, 11]. The United States Public Health Service (USPHS) criteria are used for clinically evaluating the marginal integrity of the restoration, anatomic form, marginal discoloration, axial contour, secondary caries, and biofilm [12, 13]. Radiography, because of its diagnostic efficiency, is another well-established method. Finally, SEM is more precise in assessing the quality of tooth-restoration interfaces because of its high magnifications.

The objective of this study was to evaluate longitudinally, using the 4 evaluation methods cited above, the clinical performance of restorations in deciduous molars in a patient with high caries activity using 2 different techniques, resin composite, and biologic restoration.

## 2. Case Report

A 4-year-old male patient was brought to the pediatric dental clinic of a public teaching institution for dental treatment. The child had no relevant medical history.

During clinical and radiographic examinations, extensive carious lesions were observed in the deciduous second molars. Because of the patient's poor oral hygiene, the final restorations were not placed until the patient's oral environment had improved. Temporary restorations were placed in the decayed teeth using glass-ionomer cement and the caregiver was instructed about her child's oral hygiene and dietary habits in order to control caries-promoting risk factors.

**Figure 1:**
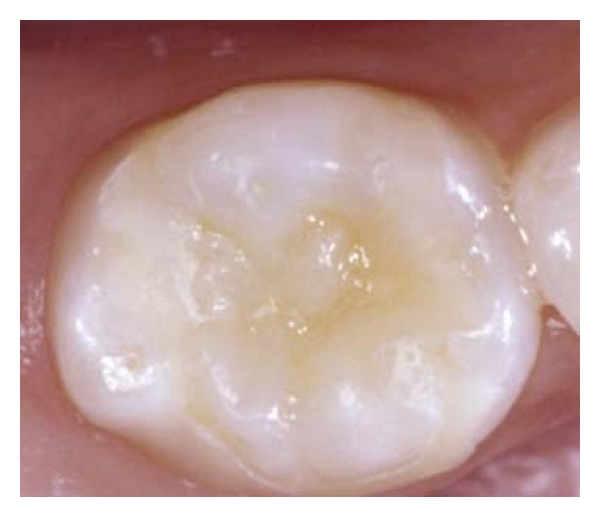
Occlusal aspect of biologic restoration immediately after completion.

Unfortunately, the patient did not show up for treatment for 10 months, and when he returned there was irreversible pulp damage in the lower right primary second molar. As a result, a pulpectomy was performed, and the root canals were filled with iodoform-based paste. Because of extensive coronal destruction involving the occlusal, buccal, and lingual surfaces, the treatment of choice was biologic restoration using a deciduous molar crown obtained from the tooth bank at the same institution (Figure 1).

After 12 months, a clinical followup of the provisional restorations was carried out. Upper right primary second molar, which had been restored with glass-ionomer cement, was treated with a formocresol pulpotomy and class I restoration with photoactivated resin composites (TPH; Dentsply, Rio de Janeiro, RJ). The other temporary molars were restored in a direct way with resin composite. Clinical visits took place every 3 months and radiographs taken every 6 months to observe the patient on a routine basis and to reinforce the caregiver's and patient's oral health preventive practices.

Five years after finishing the treatment of the lower right primary second molar, even though there was partial resorption of the root canal filling material, we did not observe any periapical lesion or clinical evidence of unsuccessful endodontics. The same applies to the upper right primary second molar, after 4 years after pulpotomy. At that time, we decided to clinically evaluate those endodontically treated teeth having the most extensive restorations. We compared the results with others obtained at the beginning of treatment (baseline). Four evaluation approaches were used: FOTI, clinical evaluation based on USPHS criteria, radiographs, and SEM (JEOL JSM 5310-SEM).

**Figure 2:**
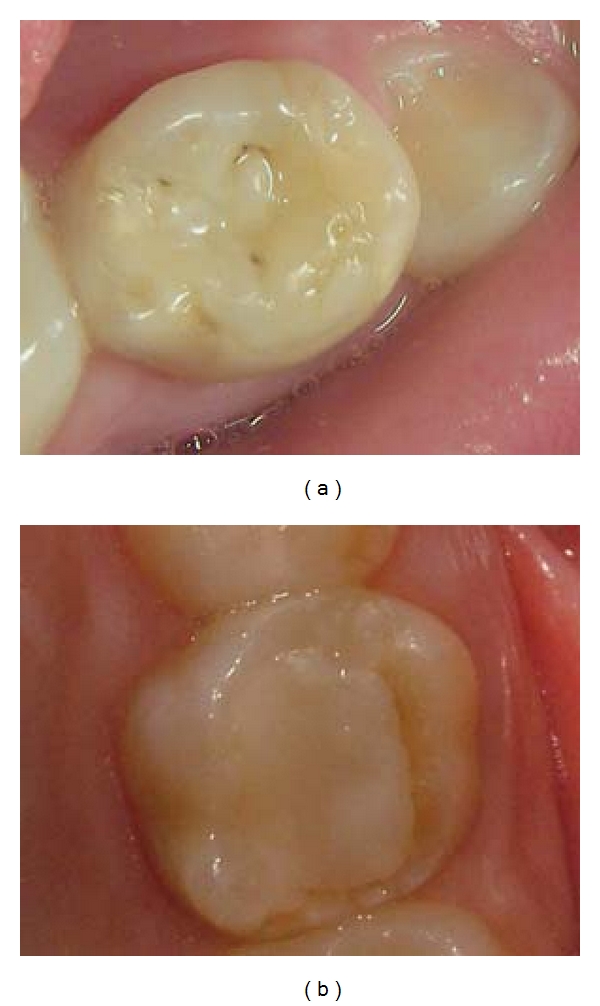
Clinical aspect: (a) biologic restoration after 5 years of followup and (b) resin composite restoration after 4 years of followup, both presenting good outcomes during clinical exam.

**Figure 3:**
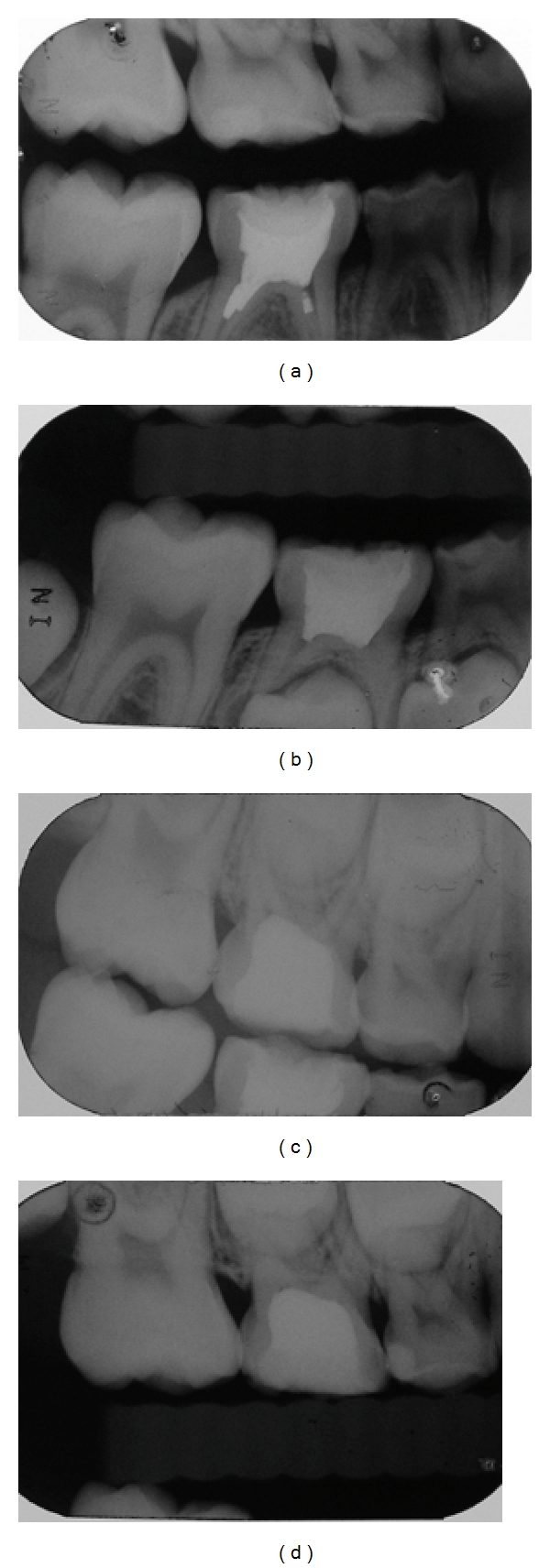
Radiographic evaluation: (a) initial and (b) final radiographs of biologic restoration; (c) initial and (d) final radiographs of resin composite restoration.

**Figure 4:**
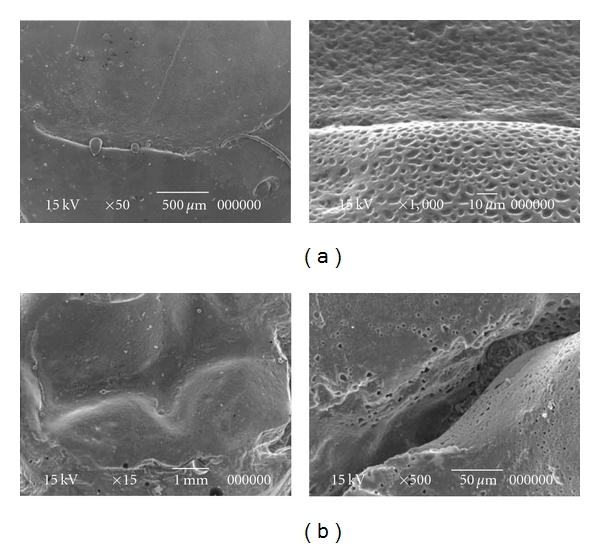
Scanning electron microscopy (SEM) at various magnifications: (a) tooth-restoration interface of biologic restoration with no evidence of microleakage after 5 years of followup and (b) tooth-restoration interface of resin composite restoration after 4 years of followup evidencing a good clinical result despite presence of a gap in 1 of the facets.

After dental prophylaxis, transillumination was performed using a photoactivating device (Heliomagic HD; Vigodent, Rio de Janeiro, RJ). The FOTI scores were established according to the shadow produced on the tooth-restoration interface according to the index created by Santos et al. [14]. The FOTI criteria were 0 (no shadow on the interface), 1 (fine shadow restricted to the enamel), and 2 (shadow reaching dentine). After this analysis, dental restoration failure was observed. This finding was further confirmed by the similarity between baseline and final results. Clinical evaluation (Figure 2) using USPHS criteria was performed by one trained investigator, and the results are shown in Table 1.  Figure 3 shows absence of microleakage according to follow-up radiographs of the 2 restorative techniques. Finally, Figure 4 shows the restoration interface of 2 teeth using an SEM. To perform this task, a Xantopren impression was transferred in epoxy resin for replication. These replicas were gold sputtered and then studied in the SEM. Although no microleakage was observed in the dental elements, a small gap was found in the tooth restored with composite.

## 3. Discussion

Despite technical advances in dentistry, there are no restorative materials having the ideal characteristics required for sealing cavity preparations. However, in view of the need for dental rehabilitation, one can find great diversity in the literature of preconized restorative materials and techniques, all aimed at treating deciduous molars with extensive coronal destruction. For Ram et al. [15], the restoration of these teeth represents a challenge, and some aspects should be taken into account to achieve satisfactory results, including natural tooth colour, durability, biocompatibility with pulp tissue, technical simplicity, and one-appointment procedure.

Several studies have shown the efficacy of metals for restorative purposes, such as silver amalgam [16] and stainless-steel crowns. However, these materials are not considered aesthetically acceptable [17]. In addition, caregivers are increasingly demanding aesthetic restorative treatment for their children [18], who in turn have also been concerned about their facial appearance. As a result, Ram et al. [15] have emphasised the great effort under way to identify a more acceptable aesthetic solution for deciduous molars.

This work showed positive results in the use of adhesive materials—as well as root canal materials—after a few years of followup. Biologic restoration was used in one of the teeth (5-year followup), whereas another tooth was restored with composite (4-year followup). Similar results were also found elsewhere [1, 6, 7]. Another important aspect is the greater tendency of restoration failure over time indicated by some authors [17, 19], an event not observed in the present case report as mentioned before. However, the follow-up time for deciduous molars is shorter than that for permanent ones due to exfoliation.

Some studies of resin composite have shown a correlation between marginal deterioration and occurrence of secondary caries due to the high sensitivity of the technique in relation to this material [20]. This finding was observed byRezwani-Kaminski et al. [6],who found secondary caries in class I composite restorations.Other studies have also reported a higher prevalence of failure in restorations involving more than one dental facet [17, 21]. In the present work, the class II direct composite restoration was shown to be clinically satisfactory, presenting a gap in only one of the facets on microscopic image analysis. On the other hand, despite involving 3 facets, the biologic restoration showed a superior clinical and microscopic outcome (no observed failure in tooth restoration interface) compared with the direct composite restoration. One can speculate that biologic restoration is less likely to suffer alterations because of the thin composite layer applied for fixation. This finding was corroborated by Ramires-Romito et al. [8], who supported the biologic restorations as a good alternative in terms of low surface erosion and optimal adaptation. However, it should be emphasised that this technique has some limitations compared with resin composites, since the practitioner must rely on their existing teeth banks and spend 2 appointments performing the procedure, while caregivers need to accept the homogenous bonding. On the other hand, because restoration adaptation is done in the laboratory, chair time is shorter—a very distinct plus in pediatric dentistry.

For evaluating restoration techniques, transillumination was shown to be advantageous due to its low cost and ease of transportation. This technique is not only well established as an effective in diagnosing carious lesions [22], but it also fulfills the objectives proposed by the present study, namely, detecting microleakage in restored deciduous teeth. Similar results were obtained by Santos et al. [14], who used this technique to evaluate microleakage in 66 deciduous molars restored with adhesive materials. Statistical analyses of that study showed that FOTI is useful in predicting microleakage; it can also be used to evaluate the clinical behavior of adhesive restorations in primary molars.

Clinical evaluation using USPHS criteria was shown to be efficient, for the results that corresponded with those from the other 3 approaches. One can state, therefore, that transillumination, clinical evaluation and radiographs are complementary. However, Vann et al. [19] have pointed out that USPHS criteria are not sensitive enough to detect early anatomic failure in restorations, a fact not observed in the present work.

Despite being relatively inaccessible to practitioners, SEM is undeniably an efficient method of indirect evaluation because of its increased magnification, allowing better visualisation of the tooth-restoration interface.

## 4. Conclusion

Despite poor biofilm control and consequent coronal destruction of some deciduous molars, the restorative techniques used in the present study were shown to be efficient after several years of followup. A higher success rate in sealing the interface tooth-restoration was obtained by the biologic bonding method.

## Figures and Tables

**Table 1:** Clinical evaluation of restorations using USPHS criteria.

USPHS criteria	Biologic restoration		Composite resin restoration	
	Baseline	After	Baseline	After
Marginal integrity	A	B	A	B
Anatomical form (wear)	A	B	A	B
Marginal discoloration	A	B	A	B
Axial contour	A	A	—	—
Secondary caries*	A	A	A	A
Plaque accumulation*	A	B	A	A

A (Alfa): clinically ideal; B (Bravo): clinically acceptable; C (Charlie): clinically unacceptable.

*A (Alfa): not present, B (Bravo): present.
